# Restricted mean survival time: an alternative to the hazard ratio for the design and analysis of randomized trials with a time-to-event outcome

**DOI:** 10.1186/1471-2288-13-152

**Published:** 2013-12-07

**Authors:** Patrick Royston, Mahesh KB Parmar

**Affiliations:** 1 MRC Clinical Trials Unit at UCL, Aviation House, 125 Kingsway, London WC2B 6NH, UK

**Keywords:** Time-to-event data, Randomized controlled trials, Hazard ratio, Non-proportional hazards, Logrank test, Restricted mean survival time, Piecewise exponential distribution

## Abstract

**Background:**

Designs and analyses of clinical trials with a time-to-event outcome almost invariably rely on the hazard ratio to estimate the treatment effect and implicitly, therefore, on the proportional hazards assumption. However, the results of some recent trials indicate that there is no guarantee that the assumption will hold. Here, we describe the use of the restricted mean survival time as a possible alternative tool in the design and analysis of these trials.

**Methods:**

The restricted mean is a measure of average survival from time 0 to a specified time point, and may be estimated as the area under the survival curve up to that point. We consider the design of such trials according to a wide range of possible survival distributions in the control and research arm(s). The distributions are conveniently defined as piecewise exponential distributions and can be specified through piecewise constant hazards and time-fixed or time-dependent hazard ratios. Such designs can embody proportional or non-proportional hazards of the treatment effect.

**Results:**

We demonstrate the use of restricted mean survival time and a test of the difference in restricted means as an alternative measure of treatment effect. We support the approach through the results of simulation studies and in real examples from several cancer trials. We illustrate the required sample size under proportional and non-proportional hazards, also the significance level and power of the proposed test. Values are compared with those from the standard approach which utilizes the logrank test.

**Conclusions:**

We conclude that the hazard ratio cannot be recommended as a general measure of the treatment effect in a randomized controlled trial, nor is it always appropriate when designing a trial. Restricted mean survival time may provide a practical way forward and deserves greater attention.

## Background

Most randomized controlled trials (RCTs) with a time-to-event outcome are designed and analyzed with a target hazard ratio (HR) for the treatment effect in mind. By convention, the HR is usually taken as the hazard function in the research arm divided by that in the control arm, with values < 1 representing a ‘positive’ treatment effect. In advanced cancers with a mortality outcome, for example, a popular choice of target HR is 0.75. This implies a reduction of 25 percent in the instantaneous mortality rate at all times after randomization. According to a standard sample size calculation based on the logrank test, about 510 events are needed to attain power 90 percent to detect such a treatment effect at a two-sided significance level of 5 percent in a trial with equal allocation to control and research arms.

For a single HR to make scientific sense, we must assume that proportional hazards (PH) of the treatment effect holds, at least approximately. We have argued previously [1] that when the PH assumption fails, it is misleading to report the treatment effect through the estimated HR, since it depends on follow-up time. A simple example of departure from PH occurs when one group is assigned to immediate surgical treatment and the other to medical treatment. Suppose time of randomisation is the origin of the survival time. When surgery increases short-term mortality but confers long-term benefit on the survivors – a reasonable alternative hypothesis – PH does not apply and the HR is a misleading and inappropriate summary.

More technically, we are unconvinced by papers such as Schemper *et al *[2] where an overall estimate of the HR is regarded as an average of time-dependent HRs over the event times, nor by proposed variants based on different and arbitrary weighting schemes. The main issue is that an average HR is uninterpretable. Under PH, for example, the HR can usefully be applied to the survival function in the control arm to obtain an impression of the survival curve in the research arm. When PH is breached, this property no longer holds. Furthermore, the HR depends on the follow-up time.

It has become apparent in some recently reported trials, e.g. IPASS [3] and ICON7 [4], that gross breaches of the PH assumption can and do occur—even to the extent of observing crossing survival curves, where a local estimate of the log HR changes sign over time. Non-PH may be due to different biological modes of action of the treatments being compared, or as identified in IPASS, to the presence of differentially responsive sub-populations.

As noted before [1], we are dissatisfied with the HR as a universal summary measure. For example, even when PH holds, the HR is not as meaningful clinically as some type of difference in average survival times or proportions at a fixed time-point, obscuring the absolute difference between the treatments and failing to convey the clinical value of a treatment. (By ‘survival time’ we mean generic time to event, for whatever event is of interest.) Furthermore, early stopping rules that assume PH can generate inappropriate decisions if the HR later changes substantially. Also, no single summary of HR or risk difference can adequately describe cases in which the treatment effect changes in direction as follow-up increases.

In our earlier paper [1], we suggested an approach to the analysis of an RCT in which the PH assumption is breached. We proposed to estimate and report the restricted mean survival time (RMST) [5], expressing the treatment effect as the difference in RMST between the randomized arms at a suitable follow-up time, *t*^∗^. We constructed confidence intervals through the standard error of the difference in RMST. Further experience with the RMST measure in a larger number of trials has given us the impression that when the PH assumption is approximately satisfied, the test of the null hypothesis based on RMST difference often has operating characteristics similar to the logrank test. Specifically, the significance level and power of the two tests appear to be similar.

An advantage of the RMST is that it is valid under any distribution of the time to event in the treatment groups, of which PH models are a (small) sub-class. Furthermore, it is readily interpretable as the ‘life expectancy’ between randomization (*t* = 0) and a particular time horizon (*t* = *t*^∗^). Owing to the current dominance of the HR and its presumed time independence, trial reports often ignore the possibility of non-PH and typically place little emphasis on the extent of follow-up, which should be a key aspect of the trial design and analysis. For example, a treatment effect can exhibit PH in the short term but non-PH over a longer period (e.g. the GOG111 trial, see Reference [1]). It is particularly important to ensure sufficient follow-up when there may be good biological or other reasons to expect the effect of a treatment to vary over time. The primary estimate of the RMST is specifically aligned to a chosen *t*^∗^ and this must be made explicit. Obviously, though, as part of the analysis, the treatment effect can be explored over a range of alternative *t*^∗^ values.

In a previous report [6], we described the implementation of a general method, Assessment of Resources for Trials or ART, for designing a trial allowing for possible non-uniform accrual rates, non-proportional hazards, loss to follow-up and cross-over of patients between treatment arms. With ART, the treatment effect is assessed using the logrank test, irrespective of whether the design assumes PH or not. A central tool in the approach is the realistic representation of the survival function in each trial arm as a piecewise exponential distribution. Recruitment and follow-up time is divided into several ‘periods’ of equal duration. Recruitment is carried out during a subset of these periods, and all recruited patients are followed up for the remaining periods. The accumulated data are analyzed when the necessary numbers of events have accrued. In addition to the usual signficance level and power, the researcher specifies the survival function in the control and research arm at the ends of selected periods. The piecewise constant hazard function is inferred from these values. At its simplest, the method accepts a single exponential distribution in each of the control and research arms, characterized by a single, constant hazard or equivalently by the median time to event. Considerable flexibility is available with a piecewise exponential model, allowing a wide range of survival distributions appropriate to the disease in question to be accommodated.

In this paper, we consider replacing a logrank-based sample size calculation and presentation of results with one based on RMST and its difference between trial arms. The difference in RMST is determined by the survival functions specified in the control and research arms through piecewise exponential distributions, exactly as in ART. Part of the paper is concerned with technical details of the calculation of RMST and its standard error under a piecewise exponential model. The results are needed in the sample size calculations. We report a small simulation study comparing the significance level and power of the logrank and RMST tests under a piecewise exponential model with non-proportional or proportional hazards, incorporating staggered entry of patients and varying length of recruitment and follow-up.

In the section ‘Restricted mean survival time (RMST)’, we describe the RMST and the corresponding standard deviation (RSDST) in general terms and specifically for a piecewise exponential distribution. Section ‘A strategy for design and analysis of clinical trials discusses our proposed strategy for trial design and analysis. We describe how to do a sample size calculation for a trial using the RMST difference. We also consider the choice of suitable values of *t*^∗^ at the design and analysis stages. We also suggest an approach to assessing maturity (readiness for analysis) of accumulating trial data according to the RMST method. Section ‘Examples’ includes limited simulation studies of the significance level and power of hypothesis tests based on the RMST difference under non-PH and PH. We provide examples in real trials. Section ‘Further issues’ makes a qualitative comparison between various measures of a treatment effect and describes results of RMST and logrank analyses in four cancer trials. We finish with a discussion and our conclusions.

## Methods

### Restricted mean survival time (RMST)

#### ***Definition of RMST***

The restricted mean survival time, *μ* say, of a random variable *T* is the mean of the survival time *X* = min(*T*,*t*^∗^) limited to some horizon *t*^∗^ > 0. It equals the area under the survival curve *S* (*t*) from *t* = 0 to *t* = *t*^∗^[5,7]:

(1) $$\mu =E\left(X\right)=E\left[\text{min}\left(T,t^{*}\right)\right]=\int_{0}^{t^{*}}S\left(t\right)\text{dt}$$

When *T* is years to death, we may think of *μ* as the ‘ *t*^∗^-year life expectancy’. In a two-arm clinical trial with survival functions *S*_0_(*t*) and *S*_1_(*t*) in the control and research arms, respectively, the difference in RMST between arms, *Δ*, is given by

$$\begin{array}{lll} \Delta & =\int_{0}^{t^{*}}S_{1}\left(t\right)\text{dt}-\int_{0}^{t^{*}}S_{0}\left(t\right)\text{dt}\; & \\ & =\int_{0}^{t^{*}}\left[S_{1}\left(t\right)-S_{0}\left(t\right)\right]\text{dt}\; & \; \end{array}$$

i.e. *Δ* is the area between the survival curves.

#### ***Restricted standard deviation of survival time (RSDST)***

To compute the variance, var (*X*), of the restricted survival time *X*, we need *E* (*X*^2^):

$$E\left(X^{2}\right)=E\left(T^{2}|T\leq t^{*}\right)\mathrm{Pr}\left(T\leq t^{*}\right)+t^{*2}\mathrm{Pr}\left(T>t^{*}\right)$$

In terms of the survival function *S* (*t*), we have Pr(*T* ≤ *t*^∗^) = 1-*S*(*t*^∗^) and

$$\begin{array}{lll} E\left(T^{2}|T\leq t^{*}\right)\mathrm{Pr}\left(T\leq t^{*}\right) & =\int_{0}^{t^{*}}t^{2}f\left(t\right)\text{dt}\; & \\ & =t^{*2}\left[1-S\left(t^{*}\right)\right]\; & \\ & \;-\int_{0}^{t^{*}}2t\left[1-S\left(t\right)\right]\text{dt}\; & \end{array}$$

where *f*(.) is the density function of *T*. Hence

$$\begin{array}{lll} \;E\left(X^{2}\right) & =t^{*2}\left[1-S\left(t^{*}\right)\right]-2\int_{0}^{t^{*}}\;t\left[1-S\left(t\right)\right]\text{dt}+t^{*2}S\left(t^{*}\right)\; & \\ & =t^{*2}-2\int_{0}^{t^{*}}\text{tdt}+2\int_{0}^{t^{*}}\text{tS}\left(t\right)\text{dt}\; & \\ & =2\int_{0}^{t^{*}}\text{tS}\left(t\right)\text{dt}\; & \end{array}$$

so that

(2) $$\begin{array}{lll} \text{var}\left(X\right) & ={\text{RSDST}}^{2}=E\left(X^{2}\right)-{\left[E\left(X\right)\right]}^{2}\; & \\ & =2\int_{0}^{t^{*}}\text{tS}\left(t\right)\text{dt}-{\left[\int_{0}^{t^{*}}S\left(t\right)\text{dt}\right]}^{2}\; \end{array}$$

The restricted standard deviation (RSDST) is $\sqrt{\text{var}\left(X\right)}$.

#### ***RMST and RSDST for the piecewise exponential distribution***

Analytic results for RMST and RSDST are available when the survival time has a piecewise exponential distribution. The integrals required in (2) are tractable. Details of the calculations and the results are given in the Appendix: RMST and RSDST for a piecewise exponential distribution.

## A strategy for design and analysis of clinical trials

### The ART approach

The ART approach to trial design [6,8] is based on specifying (log) HRs and testing their difference from zero with the logrank test. It may be used to design a trial with two or more parallel groups and a time-to-event outcome. ART allows the user to specify a recruitment phase with a predefined pattern of staggered patient entry and a follow-up phase at the end of recruitment, a standard feature of sample size calculations for such trials. Furthermore, among other advanced features, ART supports designs with non-proportional hazards, which are specified according to period-specific, time-dependent HRs.

The main design characterstics of ART are as follows: 

1. Power and significance level for a logrank test of the treatment effect (e.g. 0.9 and 0.05);

2. A number *K* of notional study periods of equal length in suitable units of real (calendar) time, over which the trial is intended to run;

3. Recruitment of patients over the first *K*_1_ periods and follow-up of all accrued patients over the remaining *K*_2_ periods, with *K*_1_ > 0, *K*_2_ ≥ 0, such that *K* = *K*_1_ + *K*_2_;

4. A relative weight for the number of patients expected to be recruited in each period (since recruitment often starts slowly and picks up as the trial’s existence becomes better known);

5. Control-arm survival function specified at some or all of the *K* periods. An alternative would be to specify the survival in the research arm at the same time points;

6. Target HR(s) under the alternative hypothesis. Hazard ratios may be specified as a single overall value (proportional hazards assumption) or for individual periods (time-dependent HR). An alternative to specifying HRs would be to provide the survival function in the research arm as well as in the control arm.

The end result is a complete definition of piecewise exponential models for the data expected under the null and alternative hypotheses. The null hypothesis is that the HRs are all equal to 1. The alternative hypothesis is that they are as given (implicitly or explicitly) in step 6 above.

The ART methodology for a two-arm trial assumes that a logrank test of the null hypothesis is to be used even if the trial has been designed with a time-dependent HR. Although not explicit, the implication is that the main trial result would be reported as the HR with a confidence interval (CI). As we have already discussed, we should not necessarily accept a single HR as an adequate trial summary statistic, particularly when the trial was designed with an expectation of non-proportional hazards.

In what follows, we suggest replacing the ART sample size calculation and presentation of results with one based on RMST and testing the RMST difference between arms. Other than the need to define a suitable time horizon *t*^∗^ for RMST evaluation, the components of the design are as described above. We present the details in the next section.

### Sample size for RMST difference

The basis of our approach is the familiar comparison of two means using an unpaired *t* test. Suppose we are sampling at random from the distribution of a positively bound random variable, *T*. We sample *n*_0_ patients from the control arm and *n*_1_ patients from the research arm. The total sample size for the trial is *n* = *n*_0_ + *n*_1_. Define the allocation ratio as *r* = *n*_1_/*n*_0_.

Suppose the means and variances of *T* in the control and research arms are *μ*_0_, $\sigma_{0}^{2}$ and *μ*_1_, $\sigma_{1}^{2}$, respectively. The null hypothesis is *H*_0_ : *μ*_0_ = *μ*_1_, with $\sigma_{0}^{2}$ and $\sigma_{1}^{2}$ unspecified. The alternative hypothesis is *H*_1_ : *μ*_0_ ≠ *μ*_1_. Suppose we wish to test the null hypothesis with power *ω* at two-sided significance level *α*. Let *Δ* = 0 and *Δ* = *μ*_1_ - *μ*_0_ ≠ 0 be the difference in RMST under *H*_0_ and *H*_1_, respectively. Following, for example, Reference [9] (p. 332), the required sample size in the control arm is

(3) $$n_{0}=\frac{{\left(z_{1-\alpha /2}+z_{\omega }\right)}^{2}}{\Delta^{2}/\left(\sigma_{0}^{2}+r^{-1}\sigma_{1}^{2}\right)}$$

hence the total sample size is

$$n=\left(1+r\right)\frac{{\left(z_{1-\alpha /2}+z_{\omega }\right)}^{2}}{\Delta^{2}/\left(\sigma_{0}^{2}+r^{-1}\sigma_{1}^{2}\right)}$$

where *z*_*p*_ = *Φ*^-1^ (*p*) is the inverse standard normal distribution function at probability *p*. An approximate (typically, conservative) sample size estimate, assuming that $\sigma_{0}^{2}≃\sigma_{1}^{2}=\sigma^{2}$, is given by

$$n≃\left(1+r\right)\left(1+r^{-1}\right)\frac{{\left(z_{1-\alpha /2}+z_{\omega }\right)}^{2}}{\Delta^{2}/\sigma^{2}}$$

We see that *n* is a minimum when *r* = 1 (equal allocation) and *n* increases substantially for larger or smaller *r* (unequal allocation).

The power, *ω*, is given by

$$\omega =\Phi \left\{{\left[\frac{\Delta^{2}}{\left(1+r\right)\left(\sigma_{0}^{2}+r^{-1}\sigma_{1}^{2}\right)}\right]}^{1/2}-z_{1-\alpha /2}\right\}$$

In the standard case, the approach would be to estimate

$$\begin{array}{lll} \;\hat{\Delta } & ={\hat{\mu }}_{1}-{\hat{\mu }}_{0}\; & \\ \;\text{var}\left(\hat{\Delta }\right) & ={\left[\text{SE}\left(\hat{\Delta }\right)\right]}^{2}=\frac{{\hat{\sigma }}_{0}^{2}}{n_{0}}+\frac{{\hat{\sigma }}_{1}^{2}}{n_{1}}\; & \end{array}$$

from the data, and test

$$z=\frac{\hat{\Delta }}{\text{SE}\left(\hat{\Delta }\right)}$$

against a Student’s *t* or (in large samples) a normal reference distribution. The usual assumption is that the response variable is normally distributed $T∼N\left(\mu_{j},\sigma_{j}^{2}\right)$ in arm *j* (*j* = 0,1). The RMST context differs from this in the following ways: 

1. The response variable is the restricted time to event, *X* = min(*T*,*t*^∗^). Due to the right truncation of *T*, the distribution of *X* is strongly non-normal;

2. In trials with a time-to-event outcome, *T* is almost invariably positively skew anyway, sometimes considerably so;

3. Right-censoring of *T* affects estimation of $\hat{\Delta }$ and SE$\left(\hat{\Delta }\right)$.

Our strategy is to take the standard case described above as our starting point for RMST sample size calculations based on the ART design assumptions, and modify it as necessary. To complete the design, we then addresss the important question of selecting *t*^∗^.

### Standard error of RMST in the ART setting

Given the ART workup, the sample size calculation given in eqn. (3) implicitly requires the variance of RMST under the piecewise exponential model around which ART is based. Consider a sample *T*_1_,…,*T*_*m*_ with no censoring before *t*^∗^ (there could be censoring after *t*^∗^). The restricted times to event, *X*_*i *_= min(*T*_*i*_,*t*^∗^) (*i* = 1,…,*m*), are an independent, identically distributed sample from some distribution. Notionally, the RMST *μ* may be estimated as the sample mean $\hat{\mu }=m^{-1}\overset{m}{\underset{i=1}{\sum }}X_{i}$, and its standard error, SE$\left(\hat{\mu }\right)$, as RSDST/$\sqrt{m}$, where RSDST is the sample standard deviation.

With censoring of some observations before *t*^∗^, the RMST is estimated by integration as in (1) and the RSDST as $\sqrt{\text{var}\left(X\right)}$ in (2). We no longer expect RSDST/$\sqrt{m}$ to be an accurate estimate of SE$\left(\hat{\mu }\right)$, since it does not reflect the increased uncertainty associated with censoring.

Consider a restricted sample *X*_1_,…,*X*_*m*_ with *μ* = *E* (*X*) estimated by integration. (Note that *m* bears no relation to the number of patients required in a trial.) Write

(4) $$\text{SE}\left(\hat{\mu }\right)=\phi \frac{\text{RSDST}}{\sqrt{m}}$$

where *ϕ* is some positive scaling factor. Clearly *ϕ* ≃ 1 for samples without censoring before *t*^∗^, but otherwise *ϕ* is unknown. In exploring simulated trial data with staggered entry of patients and a fixed follow-up time, we found that *ϕ* was very close to 1 when patients were recruited over a relatively short period and followed up for a reasonably long length of time. However, *ϕ* could increase substantially when recruitment was over a longer period with shorter follow-up. This finding accords with intuition, since more censoring and hence greater uncertainty is expected in the latter case.

In general, *ϕ* in (4) must be estimated under a known (hypothesized) piecewise exponential model. We do this by Monte Carlo simulation, as follows. First, we draw a large random sample of *m* time-to-event observations from the piecewise exponential distribution of interest and determine

$$\begin{array}{l} \phi =\frac{\sqrt{m}\text{SE}\left(\hat{\mu }\right)}{\text{RSDST}} \end{array}$$

SE$\left(\hat{\mu }\right)$ is estimated by the delta method within a flexible parametric model [10,11], using the stpm2 program [12] for Stata. Estimation with a flexible parametric model is more stable than directly with a piecewise exponential model, since sparsely populated time intervals between knots can cause fitting difficulties for the latter model. Note that RSDST is a known function of the design parameters (see Appendix: RMST and RSDST for a piecewise exponential distribution) and does not need to be estimated by simulation.

Given estimates of *ϕ*_*j*_ (*j* = 0,1), we can determine the $\sigma_{j}^{2}$ in eqn. (3) through the expression

(5) $$\sigma_{j}^{2}={\left(\phi_{j}{\text{RSDST}}_{j}\right)}^{2}$$

All of *ϕ*_*j*_, $\sigma_{j}^{2}$ and *n* have ‘Monte Carlo error’ due to the simulation. To quantify Monte Carlo error, the simulation is repeated with *M* independent samples. In each of the *M* samples, *n* is determined from (3) via (5). The SE of *n* over the *M* samples is $\sqrt{(\text{sample variance of the}n\text{’s})/M}$. We choose *M* such that the SE of *n* is sufficiently small for practical purposes. Following exploration (not reported) with different choices of *m* and *M*, we suggest taking *m* = 10000 and *M*  =50 as initial defaults, but *m* and *M* can be adjusted to suit circumstances.

Note that the only components of the sample size calculation that change with recruitment (*K*_1_) and follow-up (*K*_2_) times are the *ϕ*_*j*_. An illustration of this point is given in the section ‘Examples’.

We next describe the estimation of $\hat{\Delta }$ and SE$\left(\hat{\Delta }\right)$ from trial data.

### Estimation of $\hat{\Delta }$ and SE $\left(\hat{\Delta }\right)$ in trial data

As we discussed in our previous paper [1], several methods of estimating RMST are available, including direct integration of Kaplan-Meier survival curves, a jackknife method, and flexible parametric regression modelling [10,11]. We pointed out that the direct integration of Kaplan-Meier curves may be unreliable. The jackknife method has the advantage of being non-parametric but the drawback of being relatively slow to compute. Its slowness makes it cumbersome when simulation with many replicates is needed. We therefore prefer the third method, flexible parametric modelling, which is fast and efficient.

In the context of a randomized trial, it is essential that the estimation method be predefined, i.e. not requiring the analyst to make data-dependent modelling decisions with the actual trial data. Flexible parametric models are suitable tools for the purpose, because, for example, a cumulative hazards model with 3 d.f. fitted to each treatment arm separately appears to give an adequate fit to a wide variety of survival curves. Proportional hazards is not assumed. This particular model is assumed subsequently in the present paper for both estimation and simulation purposes.

For a given trial dataset, SE$\left({\hat{\mu }}_{j}\right)$ (*j* = 0,1) may be calculated by the delta method separately in each arm. Hence

(6) $$\begin{array}{ll} \hat{\Delta } & ={\hat{\mu }}_{1}-{\hat{\mu }}_{0}\; \end{array}$$

(7) $$\begin{array}{ll} \text{SE}\left(\hat{\Delta }\right) & =\sqrt{\text{SE}{\left({\hat{\mu }}_{0}\right)}^{2}+\text{SE}{\left({\hat{\mu }}_{1}\right)}^{2}}\; \end{array}$$

A test the null hypothesis *Δ* = 0 is made by comparing $\hat{\Delta }/\text{SE}\left(\hat{\Delta }\right)$ with a standard normal distribution.

### Choice of *t*^∗^ for the design

In our earlier paper [1], we suggested reporting the RMST and its difference between trial arms, with a CI. For such an analysis, a time (*t*^∗^) for calculation of the RMST needs to be specified. The ART-based approach to trial design defines a recruitment time (*K*_1_) and minimum follow-up time (*K*_2_) sufficient for recruitment of patients and estimation of their group survival curves over the follow-up period of clinical interest. We suggest determining the design value, $t_{\text{des}}^{*}$, as the *t*^∗^ which (approximately) minimizes the required sample size, *n*, given *K*_1_, *K*_2_ and the remaining parameters. This may be done by varying *t*^∗^ over the range *K*_2_ (the shortest follow-up time for any patient) to *K*_1_ + *K*_2_ (the maximum possible follow-up time of any patient within the design), and computing *n* by simulation, as described above.

### Choice of *t*^∗^ for analysis and assessment of data maturity

When it comes to the analysis of the trial data, for various reasons the precise data structure that is obtained may differ from the design under which $t_{\text{des}}^{*}$ was calculated. For example, the assumed survival distribution may be wrong, or the pattern of recruitment and follow-up may be at variance from that expected. To maximize power, we determine *t*^∗^ for the final analysis of the data. We call this value $t_{\text{final}}^{*}$. We also address the question of how to assess data maturity, i.e. determining when the accumulating data are mature enough for the final analysis of the treatment effect.

In a trial designed under proportional hazards of the treatment effect and analysed using a logrank test, the required total number of events, *e*, is usually taken as the effective sample size. The reason is because, to a good approximation, var (logHR) is proportional to 1/*e*, so that *e* is a measure of the amount of information in the data. The cumulative data in such a trial is ‘ready to analyse’ when the observed number of events reaches *e*. Monitoring the trial for maturity is then merely a matter of updating the data periodically and counting the number of events.

How should be we apply the principle of monitoring for maturity to trials designed with an RMST outcome? The estimated variance of the treatment effect provides a way forward. Combining (3) with (6), the following relationship holds for a sample size of *n* under the alternative hypothesis that *Δ* ≠ 0 is the difference in RMST at some given *t*^∗^:

(8) $$zz^{2}=\frac{\Delta^{2}}{\text{var}\left(\hat{\Delta }\right)}$$

where *zz* = *z*_*ω*_ + *z *_1  -*α*/2_. Note that var$\left(\hat{\Delta }\right)$ is the variance of the *estimated* RMST difference at the given *t*^∗^, whereas *Δ* is a *design* value. The planned power is achieved when var$\left(\hat{\Delta }\right)\leq \Delta^{2}/zz^{2}$. To determine if the trial data are mature enough to analyse, we effectively compare the variance of $\hat{\Delta }$ estimated from the current data with the target value, *Δ*^2^/*zz*^2^. Let us define the ‘percent maturity’ of the accumulating data as

(9) $$\text{pmat}=100\frac{\Delta^{2}}{zz^{2}\text{var}\left(\hat{\Delta }\right)}$$

where var$\left(\hat{\Delta }\right)$ is the estimated variance of the RMST difference in the current data. As more data accumulate, *pmat* increases; when it reaches 100%, the data are ready for analysis (under the assumptions of the design).

Alternatively, we can invert eqn. (8) and calculate the power, *ω*_curr_, for the current data under the design assumptions as

(10) $$\omega_{\text{curr}}=\Phi \left[\sqrt{\frac{\Delta^{2}}{\text{var}\left(\hat{\Delta }\right)}}-z_{1-\alpha /2}\right]$$

Sometimes, for reasons of confidentiality of the accumulating trial data, it is desirable to estimate data maturity or power ignoring information on possible treatment effects. In the absence of censoring in (0,*t*^∗^), we have

$$\text{var}\left(\hat{\Delta }\right)=\frac{\sigma_{0}^{2}}{n_{0}}+\frac{\sigma_{1}^{2}}{n_{1}}$$

where $\sigma_{j}^{2}$ is approximately equal to the squared RSDST at *t*^∗^ in treatment group *j* and *n*_*j*_ is the sample size (*j* = 0,1). In the absence of treatment information, we make the simplifying assumption that $\sigma_{0}^{2}=\sigma_{1}^{2}=\sigma^{2}$. This could be regarded as an assumption under the null hypothesis of *Δ* = 0, since there is then no difference between treatments. We have

$$\text{var}\left(\hat{\Delta }\right)=\sigma^{2}\left(\frac{1}{n_{0}}+\frac{1}{n_{1}}\right)$$

By elementary algebra

$$\begin{array}{lll} \frac{1}{n_{0}}+\frac{1}{n_{1}} & =\frac{1}{n}\left[\left(1+r\right)+\left(1+\frac{1}{r}\right)\right]\; & \\ & =\frac{1}{n}{\left(\sqrt{r}+\sqrt{\frac{1}{r}}\right)}^{2}\; & \end{array}$$

Hence

$$\text{var}\left(\hat{\Delta }\right)=\frac{\sigma^{2}}{n}{\left(\sqrt{r}+\sqrt{\frac{1}{r}}\right)}^{2}$$

Taking *σ*^2^/*n* as an estimate of var$\left(\hat{\mu }\right),$ the variance of RMST for the entire dataset, we have

$$\text{var}\left(\hat{\Delta }\right)≃\text{var}\left(\hat{\mu }\right){\left(\sqrt{r}+\sqrt{\frac{1}{r}}\right)}^{2}$$

Finally, to apply the maturity analysis we must find $t_{\text{final}}^{*}$ This is done by varying *t*^∗^ over a grid and finding the value that maximizes (9) or (10). If $t_{\text{final}}^{*}$ exceeds *t*_max_, the largest uncensored time to event in the data, to avoid extrapolation of RMST estimates from a flexible parametric model, we suggest limiting it to *t*_max_.

## Results

### Examples

#### ***Proportional and non-proportional hazards designs***

As a source of illustration, we constructed designs with PH and non-PH treatment effects based on updated data from the GOG111 trial in advanced ovarian cancer [13]. The overall survival probabilities in the control arm at the end of years 1 through 8 post-randomization were estimated to be 0.771, 0.523, 0.342, 0.236, 0.172, 0.130, 0.100, 0.078, respectively, with corresponding control-arm hazards of 0.264, 0.385, 0.425, 0.372, 0.320, 0.280, 0.261, 0.245. The hazard ratios (research arm/control arm) were estimated to be 0.71 under PH and 0.53, 0.66, 0.74, 0.81, 0.87, 0.93, 0.96, 1.00 under non-PH.

#### Determining $t_{\text{des}}^{*}$

**Figure 1:**
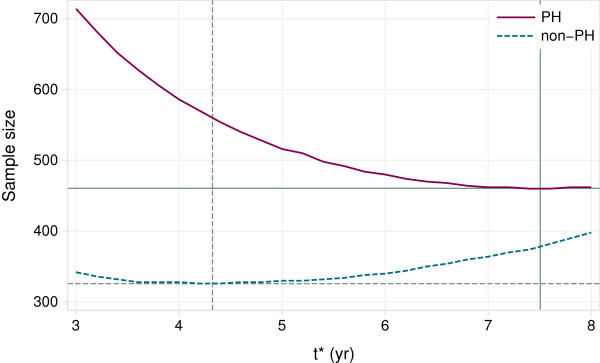
**Example of sample sizes as a function of the time horizon *t***^**∗**^** for PH (solid lines) and non-PH (dashed lines) trial designs.** The designs assume recruitment over *K*_1_ = 5 yr and follow-up over *K*_2_ = 3 yr.

We consider determining $t_{\text{des}}^{*}$ for the PH and non-PH designs just described. As an example, suppose *K*_1_ = 5 yr, *K*_2_ = 3 yr. We vary $t_{\text{des}}^{*}$ in small steps (0.2 yr) over the interval (*K*_2_,*K*_1_ + *K*_2_) = (3,8) yr and compute *n* according to eqn. (3). Figure 1 shows the resulting sample sizes for both designs. We see that *t*^∗^ and the design assumptions (PH or non-PH) both influence the sample size quite markedly. To minimize the sample size, the PH design requires a $t_{\text{des}}^{*}$ close to the maximum available follow-up time (8 yr), whereas the non-PH design needs a much smaller $t_{\text{des}}^{*}$. The $t_{\text{des}}^{*}$ values are 7.5 and 4.3 yr for the PH and non-PH cases, respectively, with corresponding sample sizes of 461 and 326.

It is apparent that the sample size does not change much for *t*^∗^ near $t_{\text{des}}^{*}$. For example, for *t*^∗^ within ±1 yr of $t_{\text{des}}^{*}$, the sample size is never larger than 9 more than the minimum, which is of no practical importance. This flexibility allows the analyst to select a preferred $t_{\text{des}}^{*}$ within a reasonably wide range without incurring a large sample size penalty.

#### ***Comparing RMST and logrank based sample sizes***

We turn to a comparison between the RMST and logrank approaches to sample size calculation. We used the ART software [8] for Stata to compute the logrank sample sizes and the numbers of events for both approaches. We used specially written Stata software that, given (*K*_1_,*K*_2_) and the other ART design parameters, finds $t_{\text{des}}^{*}$ by varying *t*^∗^ over a user-defined grid as in the previous section. It calculates the sample size by simulation according to the methods described in sections ‘Sample size for RMST difference’ and ‘Standard error of RMST in the ART setting’. The value of $t_{\text{des}}^{*}$ and the corresponding sample size were determined by smoothing the (*t*^∗^,*n*) relationship using a second degree fractional polynomial and calculating the nadir. In some cases the sample size curve over the range *t*^∗^ ∈ (3,8) yr was monotonic decreasing; we then took the optimal sample size to be for $t_{\text{des}}^{*}=8$ yr.

We investigated the impact of choices of *K*_1_ and *K*_2_ on the $t_{\text{des}}^{*}$ and *n* values, now for *K*_1_ = 1(1)7 and *K*_2_ = 8 - *K*_1_. Table 1 gives the resulting sample sizes and numbers of events for the PH and non-PH designs. For the PH designs, the sample size is similar to that for the logrank test. A $t_{\text{des}}^{*}$ at or near the maximum permissible is needed. For the non-PH designs,$t_{\text{des}}^{*}$ is around 4 yr; a markedly lower sample size is needed with the RMST approach than the logrank.

**Table 1:** Sample size calculations for hypothetical trials with proportional or non-proportional hazards of the treatment effect

**Recruit**	**Follow-up**	**PH designs**					**Non-PH designs**				
		**RMST**			**Logrank**		**RMST**			**Logrank**	
**(*K***_**1**_**, yr)**	**(*K***_**2**_**, yr)**	$t_{\text{des}}^{*}$	***n***	**Events**	***n***	**Events**	$t_{\text{des}}^{*}$	***n***	**Events**	***n***	**Events**
1	7	8	424	368	415	359	4.4	324	286	412	364
2	6	8	426	363	422	359	4.5	324	281	406	351
3	5	8	432	360	431	359	4.4	325	275	399	337
4	4	8	440	356	444	359	4.5	325	266	393	322
5	3	7.5	463	360	462	359	4.3	328	258	389	305
6	2	7.0	488	359	490	359	4.1	332	245	391	288
7	1	6.7	532	359	533	360	3.8	351	237	406	273

See the text for further details.

#### ***Operating characteristics***

We perform a small simulation study to check the power and significance level of the proposed test of RMST difference. The set-up is similar to that described in the section ‘Comparing RMST and logrank based sample sizes’, except that we vary the recruitment period (*K*_1_) over 1, 3, 5 and 7 yr, with *K*_2_ = 8 - *K*_1_. Five thousand replicates are simulated for each combination of recruitment period and null hypothesis (true or false). As before, the times to event are simulated according to a piecewise exponential distribution with staggered entry of patients at a uniform rate and RMST analysis performed with *t*^∗^ = *K*_1_/2 + *K*_2_ yr. The sample size is designed to give the test of the RMST difference power of 90 percent to reject the null hypothesis at the 5 percent level. The power and significance level of the logrank test in the non-PH and PH scenarios are also studied without altering the sample size.

The results are shown in Table 2. Two standard errors of an estimated probability of 90 and 5 percent are 0.85 and 0.62 percent, respectively. The significance levels are close to nominal for the logrank and RMST tests in both scenarios. The RMST test maintains power close to its nominal 90 percent level under both non-PH and PH. As expected from the sample sizes given in Table 1, the logrank test under non-PH is underpowered compared with planned levels. Results in Table 1 suggest that the two tests may have similar power under PH; the logrank test is slightly the more powerful.

**Table 2:** Operating characteristics of the test of RMST difference

**Model**	**Recruit**	**Follow-up**	$t_{\text{des}}^{*}$	**Sample**	**RMST test**		**Logrank test**	
	**(*K***_**1**_**, yr)**	**(*K***_**2**_**, yr)**		**size**	**Power**	**Sig. level**	**Power**	**Sig. level**
PH	1	7	8	424	89.6	4.6	90.7	4.6
	3	5	8	432	90.1	4.6	90.3	4.7
	5	3	7.5	463	90.9	5.1	90.7	4.9
	7	1	6.7	532	90.5	5.4	90.3	5.1
Non-PH	1	7	4.4	324	89.4	4.5	81.2	4.8
	3	5	4.4	325	90.0	5.0	83.2	5.2
	5	3	4.3	328	89.5	4.3	84.4	4.5
	7	1	3.8	351	91.6	4.8	83.5	5.5

Significance level and power are presented as percentages. Sample size calculations are for hypothetical trials with proportional hazards (PH) or non-proportional hazards (non-PH) of the treatment effect. Results are given for the RMST test and for the logrank test using the RMST designed sample sizes. Details are as given in Table 1.

#### ***Examples of design based on the SORCE trial in primary kidney cancer***

As a further example,we compare RMST- and logrank-based designs for SORCE, an ongoing trial in primary renal carcinoma coordinated by the MRC Clinical Trials Unit. See http://www.controlled-trials.com/ISRCTN38934710 for a summary of the trial. Only patients with an initial intermediate or poor prognosis according to the Leibovich risk score [14] are eligible. Following surgery for their kidney cancer, patients are randomized into three groups: placebo tablets, one year of treatment with tablets containing the molecular targeted agent sorafenib, or 3 years of sorafenib. We focus on the primary analysis (‘Question 1’) as defined in the trial protocol, namely ‘Does at least one year of treatment with sorafenib increase disease-free survival (DFS) compared with placebo?’.

Patients are randomized in a ratio of 2:3:3 to placebo or to the two sorafenib arms. To answer Question 1, the two sorafenib arms are combined, giving an allocation ratio of *r* = 6/2 = 3. The sample size calculation was based on the logrank test. It assumed PH with target HR = 0.75, *K*_1_ = 5 years’ recruitment with staggered patient entry and *K*_2_ = 3 years’ follow-up of all recruited patients. Power was set to *ω* = 0.9 at a two-sided significance level of *α* = 0.05. Assuming no dropout, therefore individual patients are followed up for at least 3 years and at most 8 years, depending on when they entered the trial. DFS probabilities at 1, 3, 5, 7, 10 and 13 years after surgery were estimated from values provided by Leibovich et al [14] (see Table 3). The logrank-based sample size for this design is *N* = 1656 (608 events). The possibility of increasing power by following up patients for up to *K*_2_ = 8 years, giving a total trial time of up to *K* = 13 years, was also envisaged.

**Table 3:** Design parameters for the SORCE trial

**Year**	**Trial plan**		**Hypothetical**
	**DFS prob.**	**HR (PH)**	**HR (non-PH)**
1	0.779	0.75	0.65
3	0.635	0.75	0.75
5	0.576	0.75	0.85
7	0.532	0.75	0.9
10	0.488	0.75	1.0
13	0.454	0.75	1.0

“DFS prob.” denotes survival probabilities for the disease-free survival outcome. PH and non-PH refer respectively to designs with (as per protocol) and without (hypothetical) proportional hazards of the treatment effect.

To be clear, we remind the reader that *t*^∗^ is measured in *analysis time*, with each patient’s date of entry as the origin (*t* = 0). By contrast, *K*_1_ and *K*_2_ are measured in *trial time*, i.e. in calendar time whose origins are the dates of randomization of the first and last patient, respectively.

For both logrank and RMST-based sample size calculations, we fix *K*_1_ = 5 and illustrate what happens with *K*_2_ = 3 yr (as per protocol) and with *K*_2_ = 5, 8 yr. We find $t_{\text{des}}^{*}$ as described in the section ‘Determining

$$t_{\text{des}}^{*}$$’.

We also investigate sample size for an alternative design based on non-PH of the treatment effect. The right-hand column of Table 3 shows a hypothetical but plausible pattern of time-dependent HRs, representing an initially fairly large treatment effect (HR = 0.65) which disappears (HR = 1.0) by *t* = 10 years. The sample sizes for the PH and non-PH designs are shown in Table 4.

**Table 4:** **Total sample size (*N *) and **$t_{\text{des}}^{*}$** for hypothetical trials based on the design of SORCE**

**Design**	***K***_**1**_	***K***_**2**_	$t_{\text{des}}^{*}$	**Logrank**		**RMST**	
	**(yr)**	**(yr)**	**(yr)**	***n***	**Events**	***n***	**Events**
PH	5	3	8	1656	608	1790	658
	5	5	10	1509	610	1627	658
	5	8	13	1378	612	1488	662
non-PH	5	3	5.4	1621	602	1280	476
	5	5	6.0	1803	751	1266	528
	5	8	8.0	2008	934	1488	692

Recruitment (*K*_1_) is assumed to be for 5 years in all the designs, whereas follow-up (*K*_2_) is varied over 3, 5 and 8 years.

Several features of Table 4 stand out. Not surprisingly, the sample size depends strongly on the assumed magnitude and pattern of the treatment effect. For the PH designs, the sample size is about 8 percent larger with the RMST approach than with the logrank approach. For the non-PH designs, the sample size is 27 to 42 percent larger for the logrank than the RMST approach. The RMST approach has a considerable advantage in the latter case, presumably because as the HR gets closer to 1, the power of the logrank test diminishes.

#### ***Example of maturity analysis in the MRC RE04 trial***

As an example of determining whether trial data are ready for an analysis of RMST, we consider the MRC RE04 trial in metastatic kidney cancer [15]. Following an increase in planned sample size after the start of the trial, the design involved randomization to two arms with allocation ratio *r* = 1 and a target hazard ratio of 0.8 for the research arm (triple therapy) compared with the control arm (interferon- *α* only). The main outcome measure was all-cause mortality (time to death for any reason). Based on a previous kidney cancer trial (MRC RE01), median overall survival time in the control arm was expected to be one year. The sample size required for power 90% at a two-sided signficance level of 5% was set according to ART methodology at 1100 patients and 845 events (i.e. deaths). This assumed the survival curve from the previous trial, and *K*_1_ = 4 yr, *K*_2_ = 1 yr. The sample size and events for an RMST design based on the same assumptions are 1108 patients and 848 events, with $t_{\text{des}}^{*}$ found to be 4.0 yr.

Accrual of patients was actually stopped when 1006 patients had been recruited. The trial opened for recruitment in April 2001 and closed in August 2006. The data were frozen for final analysis in September 2008, at which point 691 events (deaths) had been recorded.

**Figure 2:**
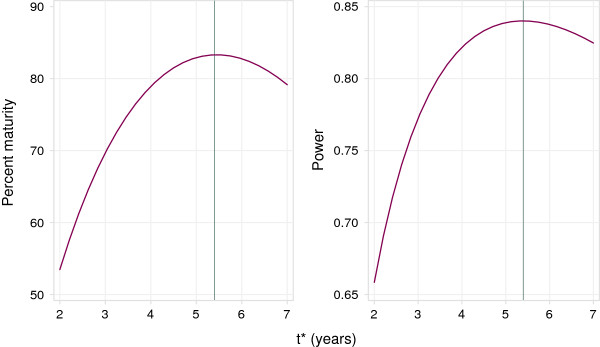
**Percent maturity (*pmat*) and power curves as a function of *t***^**∗**^** for the RE04 trial.** Vertical lines show $t_{\text{final}}^{*}$.

Since the accrual and follow-up phases were longer than originally planned, for an RMST-based maturity assessment we consider a wider range of candidates for $t_{\text{final}}^{*}$ corresponding to *K*_1_ = 2 yr, *K*_2_ = 5 yr. We estimate the survival curve from the data, ignoring treatment differences. Figure 2 shows the maturity statistic *pmat* and the power for *t*^∗^ ∈ (2,7) yr. The best choice of $t_{\text{final}}^{*}$ is 5.4 yr, at which time the maturity *pmat* = 83 percent and the power is about 0.84. The design value, *t*_*des *_*n*^∗^ = 4.0 yr, is a little low as a candidate for *t*_*final *_*n*^∗^, but may still be a reasonable option.

## Further issues

### Comparing measures of the treatment effect

Usually, the HR and its CI are reported, and often, the median survival time and/or estimated survival probabilities at fixed time points are also presented. We propose the use of RMST and statistics derived from it, as just discussed. The ‘absolute’ difference in survival (ADS) at a given time point *t*^∗^ is defined as ${\hat{S}}_{1}\left(t^{*}\right)-{\hat{S}}_{0}\left(t^{*}\right)$, where, ${\hat{S}}_{0}\left(.\right)$ and ${\hat{S}}_{1}\left(.\right)$ are the estimated survival functions in the control and research arms, respectively. How do these four measures compare on several criteria? A list of criteria and our assessments are given in Table 5. The criteria are framed in such a way that we regard ‘yes’ as advantageous and ‘no’ as disadvantageous.

**Table 5:** Comparison of four measures of the treatment effect in a trial

**Criterion**		**Measure**			
		**log HR**	**Median**^***a***^	**RMST**^***a***^	**ADS**^***a***^
1. Is easily interpreted		no	yes	yes	yes
2. Does not assume proportional hazards		no	yes	yes	yes
3. Reflects entire survival history		yes	no	yes	no
4. Is a measure of survival time		no	yes	yes	no
5. Can be used with all models		no	yes	yes	yes
6. Can be calculated in any dataset		yes	no	yes	yes
7. Does not require a time point to be specified		yes	yes	no	no
8. Does not change with extended follow-up		no	yes	yes	yes
9. Is routinely associated with a clinically meaningful time point		no	no	yes	yes

^a^The measure is the difference in the given statistic between trial arms. ADS = absolute difference in survival.

In general, difficulties with the HR and with taking the number of events as an index of the ‘maturity’ of the trial data are the following: 

1. In a very large trial, the targeted number of events can occur relatively ‘early’. However, the resulting survival functions may not be a reasonable reflection of the difference between the treatment arms over a clinically relevant time span. In an extreme case, researchers planning trials could use this approach to produce a positive result from early survival experiences, ignoring the possible later evolution of the treatment effect.

2. The same number of events may be seen in a large trial with short follow-up or a small trial with long follow-up. However, these trials are not ‘equivalent’ in the information they bear, nor in the clinical lessons that may be learned from them.

3. There are many examples where results appear to ‘change’ over time. In reality, of course, the change is an illusion caused by ignoring the time element in reporting the results.

In Table 5, RMST emerges favourably since the only ‘box’ that it fails to ‘tick’ is criterion 7. However, it may be argued that the need to define a reference time point is in fact an advantage, since it explicitly incorporates the time dimension of the trial into the results, which is often neglected as just discussed. Neither the median nor the HR do this. This aspect is reinforced in criterion 9.

The absolute difference in survival and the difference in median survival time, although often quoted, are weak because they represent only a ‘snapshot’ of the difference in survival functions. They tell us little about the previous or subsequent survival experiences. For example, the survival curves could cross at the median or at some other *t*^∗^ but still show a substantial difference in RMST at $t_{\text{final}}^{*}$.

### Examples of RMST in analysis of several trials

We compare RMST and Cox/logrank analysis in a further four MRC cancer trials: ASTEC in endometrial cancer [16] (surgery vs. standard therapy randomization), BA06 in advanced bladder cancer [17], ICON4 in ovarian cancer [18] and OE02 in oesophageal cancer [19]. In all cases, the outcome is time to death from any cause (overall survival). We have chosen these particular trials because the estimated treatment effect is approximately the same (around HR = 0.85), yet they have widely varying mortality rates. Note that in the ASTEC trial, mortality in the research arm is actually non-significantly *worse* than in the control arm. Table 6 presents some results.

**Table 6:** HR, RMST and derived statistics on survival for four randomized controlled trials in various cancer sites conducted by the Medical Research Council

**Statistic**	**ASTEC**	**BA06**	**ICON4**	**OE02**
*t *_max _(yr)	6.8	6.7	6.4	10.8
$t_{\text{final}}^{*}$ (yr)	6.1	6.7	6.3	10.8
*S *(*t*^∗^)	0.72	0.39	0.082	0.11
*N *(events ^*a*^)	1394 (188)	962 (483)	749 (417)	802 (655)
Design HR	0.75	0.75	0.75	0.75
Achieved HR	1.16	0.85	0.82	0.85
(Cox model)				
*P*-value for HR	0.3	0.07	0.04	0.03
(logrank test)				
*P*-value for non-PH	0.06	0.7	0.6	0.1
(G-T test ^*b*^)				
RMST in control	5.39	3.69	2.46	2.68
arm (${\hat{\mu }}_{0}$)				
RMST in research	5.28	4.02	2.79	3.13
arm (${\hat{\mu }}_{1}$)				
Diff. $\hat{\Delta }=$	-0.11 (0.10)	0.33 (0.18)	0.33 (0.17)	0.46 (0.26)
$${\hat{\mu }}_{1}-{\hat{\mu }}_{0}$$ (SE)				
*P*-value for	0.3	0.07	0.05	0.08
$$\hat{\Delta }$$ (RMST test)				
*P*-value for	0.07	0.4	0.7	0.04
non-PH ^*c*^				

See text for details.

^a^Events occurring in the interval (0,t^*^).

^b^Grambsch-Therneau test

^c^From a flexible parametric hazards model with a time-dependent treatment effect.

The logrank and RMST tests of the treatment effect give *P*-values with a similar interpretation. A possible exception is OE02, for which the RMST test at *t*^∗^ = 10.8 yr is not significant at the 5 percent level whereas the Cox test is significant (*P* = 0.03). However, there is evidence of a non-PH treatment effect in this trial. A hazards model with a time-dependent treatment effect suggests that the hazard ratio is below 1 near *t* = 0 and tends to approximately 1 over time.

**Figure 3:**
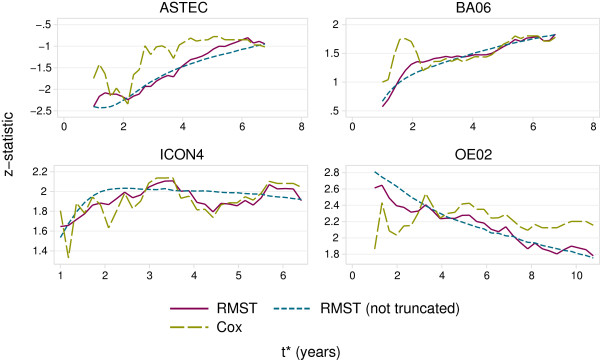
**Evolution over time (*t***^**∗**^**) of *z *-statistics for RMST (truncated, solid lines; non-truncated, short dashed lines) and Cox (truncated, long dashed lines) tests in four randomized controlled trials in cancer.**

Further insight into the behaviour of the logrank and RMST tests is provided by Figure 3. We varied *t*^∗^ in 30 equal-sized steps between 1 and $t_{\text{final}}^{*}$ years. The smooth dashed lines are for the RMST test without truncation (right-censoring) of the data. The corresponding estimates of RMST were obtained at the different values of *t*^∗^ from a flexible parametric model applied to the entire dataset. The other two lines are for the data truncated at each value of *t*^∗^. The (signed) *z*-statistic is the log HR divided by its SE from a Cox model, and the RMST difference divided by its SE from the RMST analyses. The *z*-statistics for the three methods are broadly in agreement in the ASTEC, BA06 and ICON4 trials. For OE02, however, the *z*-statistic from the Cox model is fairly constant over time, whereas for the RMST tests it diminishes steadily. Presumably the behaviour of the RMST tests is due to the non-PH pattern of the treatment effect in OE02. For the Cox test, the effect of an increasing number of events (effective sample size) may counterbalance the effect of the HR reducing over time.

A notable feature of Figure 3 is the instability of the *z*-statistic (hence *P*-value) for the tests in the truncated data, which seems greater for the Cox test. The ‘significance’ of the tests is subject to the play of chance.

## Discussion

The main advantages of our proposed method are interpretability of the RMST difference from a clinical perspective as loss of life expectancy (when the outcome of interest is mortality), and robustness of the estimator to the proportional hazards assumption. Perhaps the main disadvantages are the complexity of properly assessing data maturity (readiness for analysis) and the dependence of the test statistic on *t*^∗^. One could envisage a temptation to choose *t*^∗^ so as to obtain the ‘most significant’ result. An analysis option (not yet explored in detail) to circumvent this problem could be to derive an alternative test statistic as the minimum *z*-value for the test of RMST difference over a sensible range of values of *t*^∗^. The correct significance level of this statistic could be estimated using permutation-test methodology applied to the treatment assignment variable. However, such an analysis would be secondary to the main analysis involving a prespecified *t*^∗^.

Sample size calculations are potentially fragile, since they depend strongly on assumptions. This is seen in the SORCE example (Table 3) and in other examples. The problem is not specific to the PH assumption. It is hard to know in advance whether or not PH is a likely feature of the data to come, and if not, what a plausible pattern of time-dependent HRs might look like. In some cases, it may be reasonable to assume that a treatment effect dwindles over time, for example with treatments that are given for a relatively short period after randomization, than for it to remain constant. However, HR patterns between treatments whose modes of action differ (e.g. surgery vs chemotherapy, or targeted agent vs conventional therapy) may be hard to predict. One strategy is to compare the sample sizes arising from different plausible scenarios that include PH and non-PH examples, as we have done for the SORCE example. We would then make an informed choice based on the available evidence and on biological reasoning about the likely treatment effects. Arguably, the easiest way to define a hypothetical treatment-effect pattern is through time-dependent HRs or equivalently through the implied survival curves.

A key element of a discussion of the comparative merits of the HR and the difference in RMST as outcome measures concerns relative versus absolute effects. The HR is a relative measure which indicates neither the time to event nor the survival probability in each trial arm. Under the PH assumption, it is independent of time. The RMST difference measures the effect of treatment on the restricted survival time at some *t*^∗^. The values of RMST in each trial arm are absolute measures of survival time. This dual mode of presentation, as both a relative and an absolute measure, is an important advantage of RMST. In our view, the HR’s lack of any absolute component means the HR is incomplete as an outcome measure. It needs to be accompanied by other statistics, such as the estimated median survival times and/or the survival probabilities at specific time(s), or indeed the RMST.

The HR can seem impressively large even when the absolute effect on the time to event is small. Over-stressing the importance of apparently large relative risks has often been criticized in the medical and popular scientific literature as misleading for patients and physicians. For an example in the context of the benefit of breast cancer screening, see Reference [20] pp. 59 – 60. Here, we consider ASTEC vs. OE02 in Table 6. The absolute log HRs are almost identical yet the absolute RMST difference at *t*^∗^ = 5 years is some 3.4 times greater in OE02 than in ASTEC (0.29 vs. 0.09 years, i.e. about 3.5 months vs. 1 month). (These results for *t*^∗^ = 5 years were calculated separately; they are not given in Table 6.) The reason, of course, is that the 5-year survival probability in ASTEC is much larger than in OE02. The RMST difference of 0.09 years at *t*^∗^ = 5 years seen in ASTEC is arguably of little practical importance. In general, statistically significant differences in RMST from randomized trials may appear ‘small’, but they may be more realistic and clinically meaningful than superficially more impressive relative effects on the hazards. See also Royston et al’s [21] proposed graphical comparison of observed and imputed times to event between trial arms, which carries a similar message.

Here we have focused on RMST mainly as a potential design tool, having described the use of RMST in the analysis of trial data in a previous paper [1]. A standard approach to analysis would be to assume PH, test the null hypothesis of no treatment effect using the logrank test, and estimate the HR in a Cox model with randomized treatment as the only covariate. Adjustment for other covariates (e.g. prognostic factors) is readily incorporated. There are many options for extending the model if non-PH is detected. However, any such adaptation is likely to be data-dependent. The Cox model, whether in basic form or extended, does not readily lend itself to estimating the RMST [1]. An alternative may be to fit a piecewise exponential model with the knots used in the trial design. If too many knots are specified, the model can be over-fitted and the parameter estimates correspondingly unstable. This can happen if there are few events between a neighbouring pair of knots.

A more satisfactory analysis strategy is to use flexible parametric survival models [10-12] to estimate RMST. In summary, the PH subclass of these models incorporates a smooth estimate of the baseline log cumulative hazard function as a restricted cubic spline function of log time. The models readily lend themselves to precise estimation of RMST and RSDST and to extensions which accommodate time-dependent treatment effects (i.e. non-PH). An advantage is that their hazard functions are more realistic than those from the piecewise exponential model, since they are smooth functions of time rather than step functions. Parameter estimation by maximum likelihood is straightforward. However, to our knowledge flexible parametric models cannot be used on their own to design a trial. Piecewise exponential models are needed here, since they make it easy to specify the model in terms of survival probabilities and hazards and provide analytic expressions for the RMST and RSDST. Flexible parametric models are unsuitable for exploring hypothetical RMST values associated with a design with given hazard ratio(s) and control arm survival function.

We have described the calculation of two primary values of *t*^∗^, namely $t_{\text{des}}^{*}$ and $t_{\text{final}}^{*}$. The former is driven by the theoretical structure of the design and the latter by the trial data as recorded. It is important to note that $t_{\text{final}}^{*}$ does *not* depend on the treatment effect observed in the data, but on the designed difference in RMST and its observed variance as functions of *t*^∗^. In particular, $t_{\text{final}}^{*}$ is not selected to miminize the *P*-value for the treatment comparison. It is data-driven only with respect to the variance of the RMST difference. However, the value of *t*^∗^ at which the definitive analysis is carried out may be motivated more by clinical than statistical concerns. A graph of power or maturity against *t*^∗^, as in Figure 2, may be used to decide if the data are adequate for an analysis using some preferred value of *t*^∗^. If the data are not sufficient, it may be appropriate to extend the follow-up period and/or recruit more patients. In Figure 2, for example, *t*_*final*_*nnn*^∗^ = 5.4 yr has power 84 percent and maturity 83 percent under the PH design assumptions, whereas a lower value, say *t*^∗^ = 4 yr, might be preferred; this has power and maturity slightly reduced to about 80 and 81 percent, respectively.

An important question is whether the RMST-based sample size calculation we have proposed is robust enough to be put into practice. Tentatively, we believe it is. With designs in which PH is assumed and holds, the logrank- and RMST-based sample size requirements are similar (see Table 1), and the power for a given sample size is correspondingly similar. For designs with severe non-proportional hazards, sample sizes for logrank- and RMST-based tests can differ markedly. As always with trial design, the key assumptions of data structure and relevant parameters critically affect the required sample size, and some kind of informal sensitivity analysis should always done.

## Conclusions

In summary, we conclude that the HR can often be an inappropriate and insufficient general measure of the treatment effect in an RCT, and also that the logrank test may lack power under some patterns of non-proportional hazards. We suggest that wider exploration and use of RMST in the design and analysis of trials with a time-to-event outcome is merited.

## Appendix: RMST and RSDST for a piecewise exponential distribution

Assume that the survival time, *T*, has a piecewise exponential distribution with *k*+1 piecewise constant hazards *h*_1_,…,*h*_*k*_,*h*_*k*+1_ in a categorization (*τ*_0_ = 0,*τ*_1_], (*τ*_1_,*τ*_2_], (*τ*_2_,*τ*_3_], …, (*τ*_*k*_,*τ*_*k*+1_ = *∞*) of the time axis. The time points *τ*_1_,…,*τ*_*k*_ are known as knots. Suppose that *t*^∗^ belongs to interval (*τ*_*k*_,*τ*_*k*+1_), so that *t*^∗^ > *τ*_*k*_. In the simplest case (*k* = 0), there are no knots and we have a single exponential distribution with hazard *h*_1_, and *t*^∗^ > 0.

We wish to calculate the RMST and the RSDST at *t*^∗^. For *j* = 0,1,…,*k* the interval duration *δ*_*j*+1_ is

$$\delta_{j+1}=\left\{\begin{array}{ll} \tau_{j+1}-\tau_{j}, & j<k\\ t^{*}-\tau_{k}, & j=k \end{array}\right)$$

The cumulative hazard function *H*_*j*_ = *H*(*τ*_*j*_) at *τ*_*j*_ (*j* = 1,…,*k*) equals $\overset{j}{\underset{i=1}{\sum }}h_{i}\delta_{i}$. Let *h*_0_ = *H*_0_ = 0. The survival function for *t* ∈ (*τ*_*j*_,*τ*_*j*+1_] (*j* = 0,1,…,*k*) is

$$S_{j+1}\left(t\right)=e^{-H_{j}}e^{-h_{j+1}\left(t-\tau_{j}\right)}$$

For example, the survival function for *t* ∈ (0,*τ*_1_] is $S_{1}\left(t\right)=e^{0}e^{-h_{1}\left(t-0\right)}=e^{-h_{1}t}$, as expected.

The integrated survival function from 0 to *t*^∗^ > *τ*_*k*_ (i.e. the RMST) is given by

$$\mu =\int_{0}^{t^{*}}S\left(t\right)\text{dt}=\overset{k}{\underset{j=0}{\sum }}\int_{\tau_{j}}^{\tau_{j}+\delta_{j+1}}S_{j+1}\left(t\right)\text{dt}$$

Also

$$\begin{array}{lll} \int_{\tau_{j}}^{\tau_{j}+\delta_{j+1}}S_{j+1}\left(t\right)\text{dt} & =\int_{\tau_{j}}^{\tau_{j}+\delta_{j+1}}e^{-H_{j}}e^{-h_{j+1}\left(t-\tau_{j}\right)}\text{dt}\; & \\ & =e^{-H_{j}}\int_{0}^{\delta_{j+1}}e^{-h_{j+1}u}\text{du}\; & \\ & =\frac{e^{-H_{j}}}{h_{j+1}}\left(1-e^{-h_{j+1}\delta_{j+1}}\right)\; & \\ & =e^{-H_{j}}B_{j+1}\; & \end{array}$$

where for *j* = 0,…,*k *

(11) $$B_{j+1}=\frac{1-e^{-h_{j+1}\delta_{j+1}}}{h_{j+1}}$$

Thus the RMST on (0,*t*^∗^) is given by

$$\mu =\int_{0}^{t^{*}}S\left(t\right)\text{dt}=\overset{k}{\underset{j=0}{\sum }}e^{-H_{j}}B_{j+1}$$

We also need the expectation $E\left(X_{j}^{2}\right)$ of *X*^2^ in the interval (*τ*_*j*_,*τ*_*j*+1_] or (*τ*_*k*_,*t*^∗^], which is

$$\begin{array}{lll} E\left(X_{j}^{2}\right) & =2\int_{\tau_{j}}^{\tau_{j}+\delta_{j+1}}S_{j+1}\left(t\right)\text{tdt}\; & \\ & =2\int_{\tau_{j}}^{\tau_{j}+\delta_{j+1}}te^{-H_{j}}e^{-h_{j+1}\left(t-\tau_{j}\right)}\text{dt}\; & \\ & =2e^{-H_{j}}\int_{0}^{\delta_{j+1}}\left(t+\tau_{j}\right)e^{-h_{j+1}t}\text{dt}\; & \\ & =2e^{-H_{j}}\left[\int_{0}^{\delta_{j+1}}te^{-h_{j+1}t}\text{dt}+\tau_{j}\int_{0}^{\delta_{j+1}}e^{-h_{j+1}t}\text{dt}\right]\; & \\ & =2e^{-H_{j}}\left(A_{j+1}+\tau_{j}B_{j+1}\right)\; & \end{array}$$

where *B*_*j*+1_ is as given in (11) and

$$\begin{array}{lll} A_{j+1} & =\int_{0}^{\delta_{j+1}}te^{-h_{j+1}t}\text{dt}\; & \\ & =\frac{1}{h_{j+1}^{2}}\left[1-e^{-h_{j+1}\delta_{j+1}}\left(1+h_{j+1}\delta_{j+1}\right)\right]\; & \end{array}$$

Hence

(12) $$\begin{array}{lll} E\left(X\right) & =\overset{k}{\underset{j=0}{\sum }}e^{-H_{j}}B_{j+1}\; & \\ E\left(X^{2}\right) & =2\overset{k}{\underset{j=0}{\sum }}e^{-H_{j}}\left(A_{j+1}+\tau_{j}B_{j+1}\right)\; & \\ \text{var}\left(X\right) & =E\left(X^{2}\right)-{\left[E\left(X\right)\right]}^{2}\; \end{array}$$

For a single exponential with hazard *h*, we have *k* = 0, *τ*_0_ = 0, *δ*_1_ = *t*^∗^, *h*_1_ = *h* and therefore

$$\begin{array}{lll} A_{1} & =h^{-2}\left[1-e^{-ht^{*}}\left(1+ht^{*}\right)\right]\; & \\ B_{1} & =h^{-1}\left(1-e^{-ht^{*}}\right)\; & \\ \mu & =E\left(X\right)=B_{1},\; & \\ \sigma^{2} & =\text{var}\left(X\right)=2A_{1}-B_{1}^{2}\; & \end{array}$$

## Abbreviations

ART: Assessment of resources for trials; CI: Confidence interval; HR: Hazard ratio; MRC: Medical Research Council; non-PH: Non-proportional hazards; PH: Proportional hazards; RCT: Randomized controlled trial; RMST: Restricted mean survival time; RSDST: Restricted standard deviation of survival time.

## Pre-publication history

The pre-publication history for this paper can be accessed here:

http://www.biomedcentral.com/1471-2288/13/152/prepub
